# Improving eye-tracking calibration accuracy using symbolic regression

**DOI:** 10.1371/journal.pone.0213675

**Published:** 2019-03-15

**Authors:** Almoctar Hassoumi, Vsevolod Peysakhovich, Christophe Hurter

**Affiliations:** 1 DEVI, French Civil Aviation University - ENAC, Toulouse, France; 2 DCAS, ISAE-SUPAERO, Université de Toulouse, Toulouse, France; Central South University, CHINA

## Abstract

Eye tracking systems have recently experienced a diversity of novel calibration procedures, including smooth pursuit and vestibulo-ocular reflex based calibrations. These approaches allowed collecting more data compared to the standard 9-point calibration. However, the computation of the mapping function which provides planar gaze positions from pupil features given as input is mostly based on polynomial regressions, and little work has investigated alternative approaches. This paper fills this gap by providing a new calibration computation method based on symbolic regression. Instead of making prior assumptions on the polynomial transfer function between input and output records, symbolic regression seeks an optimal model among different types of functions and their combinations. This approach offers an interesting perspective in terms of flexibility and accuracy. Therefore, we designed two experiments in which we collected ground truth data to compare vestibulo-ocular and smooth pursuit calibrations based on symbolic regression, both using a marker or a finger as a target, resulting in four different calibrations. As a result, we improved calibration accuracy by more than 30%, with reasonable extra computation time.

## Introduction

Displaying one or many markers on a screen, during an eye tracker calibration, is a commonly used method for tracking user gaze [1, 2]. One of the most broadly used approaches is that of the standard 9-markers, where a participant looks intently at all markers displayed on a screen sequentially for a few seconds [3–5].

A substitute for the standard 9-markers is the smooth pursuit calibration (denoted SP in the sequel), in which the calibration is processed with one marker following a predefined path and covering a predefined area of the screen. SP calibration works with the tacit assumption that users will find the tracking task pleasing and that more calibration data will be collected [1]. However, additional parameters need to be taken into consideration, such as the speed of the moving target which is an important modality of the success of the calibration [6, 7]. The higher the speed, the more difficult it will be for our eyes to catch up.

In the same vein, another approach is the vestibulo-ocular reflex (VOR) calibration procedure, in which the user is asked to fixate on a static marker while turning or rotating the head. With this method, it is obvious that the constraint of keeping the user’s head still is no longer a concern. The Vestibular reflex enables humans to maintain objects of interest in the fovea during head movements. Consequently, vestibular movements are considered to be more flexible and user dependent than SP movements. Recent works [1, 8] have shown, in separate studies, that the two last mentioned methods (SP, VOR) offer some unique advantages over the standard 9-markers calibration. In particular, they allow the collection of continuous and large amounts of data. [9] investigated the time required to obtain high calibration accuracy by comparing SP and VOR calibrations over short and long time periods. Although eye tracking literature is replete with novel calibration methods, relatively limited progress has been made toward evaluating the regression models in recent calibration procedures. They are mostly based on a polynomial regression computation [10] and little work has investigated alternative techniques [11]. This paper partially fills this gap by providing a new calibration computation method based on symbolic regression. We evaluate this approach and compare with recent methods based on the accuracy of gaze estimation results.

Instead of considering a polynomial transfer model between input and output records defined by the designer, symbolic regression will seek for an optimal model between a set of different types of predefined functions and their combinations (sin, log, polynomial, etc.). This type of computation opens new perspectives in terms of flexibility and accuracy.

The contribution of this paper is threefold. First, we investigated to what extent symbolic regression allow an improvement of existing calibration techniques accuracy. Secondly, we validated this technique on ground truth data collected during a user study which aimed to compare smooth pursuit and vestibulo-ocular calibration methods. After that, we assessed the effect of symbolic regression on calibrations based on marker and finger stimulus. The two studies delved empirically into how the type of calibration procedure impacts gaze estimation accuracy. We completed 48 (12 × 4) trials with 12 participants, and investigated 4 different calibration procedures (SP using Marker, SP using Finger, VOR using Marker and VOR using Finger). We performed a thorough analysis of the collected data, propose an assessment of their accuracy and recommend design guidelines for the calibration time required. Comparisons between existing polynomial regressions and the symbolic regression are given. Overall, the calibration accuracy has been improved by more than 30%, with an extra computation time. The paper structure is as follows: we first provide a review of existing calibration techniques. Then, we review existing symbolic regression principles and their usages. Next, we detail the investigations of the experimental study, followed by detailed results. Finally, we discuss our findings and results, and give possible improvements and directions for future work.

## Related work

Different approaches have been explored to propose alternative calibration methods with better accuracy and flexibility. As an example, [12] performed a 5-points calibration using a SMI iView X HED 4 system to estimate gaze positions of one hundred and forty participants. Some studies undertook to inspect more fixed targets, up to 18 markers calibration in [13] and 45 markers in [14]. Others focused on reducing calibration time by changing and testing different layouts [10]. The standard fixed-target calibration methods are generally considered as effective calibration, particularly with respect to region coverage. However, it was found that some participants had difficulties with fixation based calibration (e.g. autistic children and infants). For such subjects, adequate methods must be considered to tolerate the effect of mental effort and maintain concentration during calibration. To address this issue, [15] considered a calibration routine based on smooth pursuit eye movements.

Prior studies [1, 8, 9, 16] have mentioned the importance of calibration routines based on moving targets unlike the fixed-target calibration, in order to optimize accuracy, collect more data and accelerate the process. [16] used smooth pursuit for their calibration technique by setting a predictable trajectory followed by the marker and a different regression technique compared to prior works. They used an Archimedean spiral trajectory with constant linear velocity (6.4°/sec), circumventing the problems raised by the trajectory used in [1]: following only the borders of the rectangle may not help retrieving the interior points. In addition, even if the data is processed seamlessly on the rectangle’s borders, the abrupt change in direction of the moving target on the rectangle’s corners may induce errors. This is particularly noticeable when dealing with high-speed moving targets. Trajectories with smooth transitions can help alleviate this phenomenon. Consequently, [1] considered reducing the speed of the moving target when approaching each corner.

In order to find a mapping function that produces gaze estimation from eye position, [16] applied a quadratic regression. They validated their method with 49 evaluation points and corrected the residuals. The results showed that the Root Mean Square Error from the non-truncated data of the smooth pursuit calibration was 0.838° (*SD* = .278, 27 seconds calibration time) compared to the 9-point calibration which gave 1.388° (*SD* = .963, 23 seconds calibration time). The authors truncated the smooth pursuit calibration data to consider similar time to the 9-point calibration for proper comparison and obtained an error of 0.913° (SD = 0.272). Results on both X and Y axes were given in their paper. Using a linear mixed model analysis, they found a significant difference between the 9-point calibration and both smooth (p = .02) and truncated smooth (p = .04) calibrations. In [17], the authors investigated collecting calibration points while following a supervisor’s thumb relocated at five different positions (moving target) and compared this procedure against an approach consisting of a user looking at a fixed point and moving her head in an asterisk-like trajectory. Approximately 20 calibration points were gathered and an offline calibration computation gave a mean error of 0.83°. [18] modified the calibration method and posed two visual markers on the PC screen. They proposed a method in which the user fixates on two separate visual markers on the screen sequentially. The visual marker is shown on the screen and moves from a position P1 to a position P2 onward and backward. The user is asked to fixate on the marker during the whole movement, making a smooth pursuit eye movement. More recently, [19] attempted to find the best terms that can be used to construct the mapping model, however, they stated that for polynomials up to 3 degrees, it may become challenging to check all possible sets. Therefore, they provide an open, extendable software (ETCAL) in which they implemented a heuristic algorithm that served to reduce the number of models to explore based on genetic programming, however, finding the best solution was not guaranteed, the computational time required to find a good model was not provided by the authors and they did not consider recent methods based on vestibulo-ocular calibration. [20] gives different calibration techniques based on polynomial regression. None of this prior work has fully investigated the impact of symbolic regression on eye tracking calibration accuracy, more specifically on smooth pursuit and vestibular movements calibrations. Additionally, it is worth noting that a huge number of different calibration procedures are being proposed by the community in order to find the most accurate gaze estimation. We notice that nearly all new proposed calibration procedures focus on the path followed by the marker or finding the best pupil center position, but omit to search the best polynomial model that provides higher accuracy. They use the traditional mapping model derived from the studies involving 9-points calibration procedures. In contrast to the standard 9-points calibrations, these new types of calibration procedures (e.g. smooth pursuit) allow obtaining more data points, therefore, a better algorithm could benefit from the large calibration dataset to provide more accurate models. Moreover, since the mapping model is not fixed in advance, every new calibration procedure using complex non-linear target paths, e.g. the Archimedean spiral trajectory used in [16], could build on this approach to retrieve the best mapping model.

## Gaze estimation using symbolic regression

Finding the underlying cause of higher residuals after polynomial interpolation has long been an objective in eye tracking calibration [21, 22]. The standard fixation calibration with more than nine visual stimuli, smooth pursuit and vestibulo-ocular calibrations were used and helped to collect more pupil-target tuples. [23] implemented a post hoc correction technique to clean up errors after the calibration has been performed. While those techniques are promising, the gaze estimation methods used were based on finding the best parameters of a predefined polynomial function model that infers eye features to gaze coordinates. The function model is generally a bivariate bilinear second-order model and is defined by the developer, imposing prior assumptions. Recently, Gaussian processes regression was provided as an alternative to polynomial interpolation [11]. This technique is non-parametric, and different algorithms can be used to estimate its models. Genetic algorithms can be exploited to optimize Gaussian process models [24].

In this paper, we introduce symbolic regression for gaze estimation to the community. The benefit of this method is that the model of the function is not given in advance. Instead, the algorithm searches for the appropriate model, along with its parameters, that infers the eye features to gaze coordinates using genetic programming. Symbolic regression has been used to find explicit models on financial data [25], economic decision-making [26], and various problem using non-linear multidimensional data were solved using genetic programming. More details can be found in [27]. Overall we show that symbolic regression can better fit gaze estimation functions detection and reduce the mean absolute error. The results of our experiments showed that the functions obtained through symbolic regression give improved results compared to common polynomial regression approaches.

### Motivation

Recently, model-based gaze estimations have gained a special interest in the community [28, 29]. They have proved to offer a high degree of accuracy. However, while these approaches seem promising, they can provide unexpected results in different scenarios. A recent study, based on synthetic images [30], showed that even though model-based gaze estimation can reach positive results, its accuracy is not stable in every situation. More specifically, it has been shown that its accuracy may decrease in relation to the refractive strength of eyeglasses. In the same study, the authors did not find any significant effect on accuracy when using standard polynomial regression. This clearly indicates that regression-based gaze estimation may still produce some benefits compared to model-based gaze estimations.

Earlier studies have compared different polynomial function models to define which gives the best results in term of accuracy. [10] examined how the polynomial structure affects estimation results by comparing over 400,000 models including models with an expression up to fourth order. The conclusion of the results is that, for any of the configuration, no model is better than the rest. However, recommendations for the appropriate model may be given.

Thereby, having a means of automatically obtaining the model of the function may be of benefit in eye tracking systems calibration.

### Definitions

Symbolic regression is the automated searching of a function model, along with its intrinsic parameters, that infers estimation of output values from input values [31]. This method relies on Genetic algorithms (GA), more specifically genetic programming [27] which searches and modifies, the best solutions that define a problem among a population of individuals. For each solution, multiple individuals are randomly picked, modified and mutated to represent the new population to explore.

Theoretically, given a set of *n* collected observations of the form {(*x*_1_, *y*_1_), …, (*x*_*n*_, *y*_*n*_)} such that *x*_*i*_ represents the eye features vector (pupil center coordinates in eye camera frame) of the *i*-th *k*-dimensional observation and *y*_*i*_ is its corresponding target (i.e the center of the marker in the world camera image), the algorithm seeks to find a set of models *F* = *f*_1_, *f*_2_, ‥that best fit the dataset:

$$\begin{array}{c} f_{p}:|\begin{array}{ccc} X & ⟶ & Y\\ x & ⟼ & f_{p}\left(x\right) \end{array} \end{array}$$ (1)

where X is the set of the pupil positions observed during the calibration and Y the output observations, namely, the center of the target in the world camera frame. The models are generated by combining and modifying the individuals of a predefined population. Therefore different models are obtained and each model projects the *k*-dimensional vector *x* into an output value $\hat{y_{i}}=f_{p}\left(x_{i}\right)\;.\;f_{p}$ is selected as the best model if its residuals, defined as follows, is the smallest:

$$\begin{array}{c} \hat{\theta }=\underset{\theta }{\text{arg}\;\text{min}}\{\begin{array}{c} \sum_{1}^{n}|\hat{y_{i}}-y_{i}| \end{array}\} \end{array}$$ (2)

Where $|\hat{y_{i}}-y_{i}|$ is the absolute difference between the estimated and the recorded target position, called the absolute error.

However, to make the computation less costly, the set of individuals that form the search space must be relatively small. In our study, we used standard functions including constant, linear, quadratic, cubic polynomials, trigonometric, logarithmic (log) and exponential (exp) functions, along with the most commonly used operators: addition (+), subtraction (-), multiplication (x) and division (/).

### Algorithm process

**Preparation**: The outcome of a Symbolic function identification (Symbolic Regression) is highly dependent on the preparation phase. Typically, the preparation phase requires the user to provide the data, the primitive functions (individuals), the fitness measurement and the termination criterion.

In eye tracking calibration, the data consists of the independent variables usually obtained from the user’s eye features such as the pupil center locations in the camera image, the pupil-center-corneal-reflection vectors, the pupil diameter, size or contour, and the dependent variables, i.e. the positions of the target center.

The primitive functions involve arithmetic, logical operations and domain specific functions.

The fitness measurement delineates the metric that serves to evaluate the quality of an extracted model. It provides a measurement of the criterion on which the algorithm selects the best model. An example of a fitness measurement used by [32] is the mean absolute error:

$$\begin{array}{c} MAE=\frac{1}{n}\sum_{i}^{n}|{\hat{y}}_{i}-y_{{}_{i}}| \end{array}$$ (3)

Where *y_i_* represents the target center positions, ${\hat{y}}_{i}$ is the estimated target center positions obtained from an extracted model, and *n* is the number of observations in the training set.

The termination criterion is used to stop the algorithm after a certain delay, for example, the algorithm could be stopped after two seconds. Otherwise, the algorithm could be implemented so that it stops after a good model is found, i.e, when the fitness measurement drops below a certain threshold (e.g., *MAE* < 0.2) or when a defined number of generations have been explored [25].

**Fig 1:**
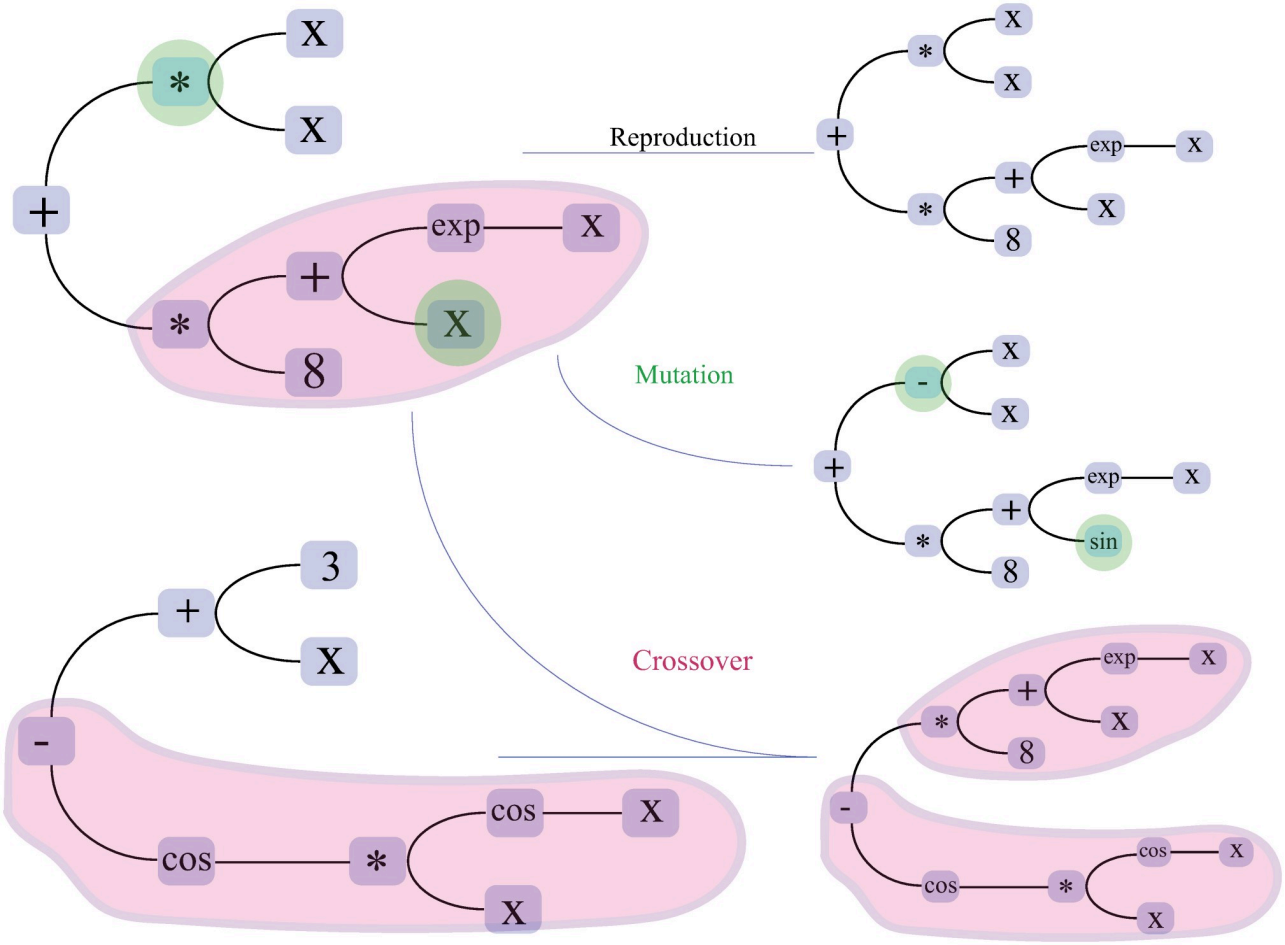
The operations for finding new models. Two models (left) are exploited to generate new models using reproduction (in blue), mutation (two elements are modified—Green) and crossover (two branches are combined—Red) operations.

**Operations**: The generated models consist of a connection of variables representing the pupil features, linked by the primitive functions and represented by a tree. The node of the three are the functions and the operators. The leaves are the pupil features and constants (Fig 1).

To find a new model, the current generated models, as shown in Fig 1, are altered and modified using genetic operations such as reproduction, mutation and crossover. Each operation is specified with a probability of using it to alter the current trees [27].

1. *Reproduction*: the reproduction operation is an adaptation of the Darwinian reproduction and survival theory [33]. It involves selecting, copying and reusing certain models of the current generation in order to maintain their survival in the next generation.
2. *Mutation*: the mutation operation modifies the current tree by changing the nodes and/or the leaves using the defined functions, operators and variables. It may consist in adding a variable, deleting a node, changing an operator (e.g. multiplication to addition).
3. *Crossover*: this operation leverages parts of two parent models to extract a new model.

**Execution**: Initially, the algorithm generates blind random models composed of the provided primitive functions, then iteratively transforms those models into more appropriate ones based on the fitness measurement. The initial randomly generated models are generally of poor quality. They are used as a starting points to derive better models.

### Examples

This section presents two different examples of how symbolic regression detects models based on the operators, population of basic functions given as input and the data obtained during a calibration procedure. We could assume we are trying to find the cubic model of the form *f*(*x*) = *x*^3^ + 8*x*^2^ + 10*x* − 427. From this model, 30 pairs (*x*_*i*_, *y*_*i*_) are retrieved where *y*_*i*_ = *f*(*x*) and *x*_*i*_ are normally distributed in [0, 100]. This dataset is given as input to the algorithm to detect the model *f*(*x*) without knowing its form beforehand. The population used consists of a list of individuals *I* = {*x*, cos, sin, log, exp, constant c∈R} and a list of operators *O* = {−, +, *, /}. At the beginning, the algorithm starts with a random solution *f*_0_(*x*) = 42 comprising one leaf. After 200 milliseconds, the best polynomial found *f*_200_(*x*) gave a mean absolute error (MAE) of 0.078 and is similar to *f*(*x*):

$$\begin{array}{c} f_{200}\left(x\right)=9.97x+8x^{2}+x^{3}-427 \end{array}$$ (4)

To investigating the effect of the algorithm on the data size, we provide the results for 3 different samples of size *n*1 = 75, *n*2 = 150, *n*3 = 200. The results are given in Table 1 and simulate the size of respectively 2.5 seconds, 5 seconds and 6.65 seconds calibration data with a 30 frames/second camera.

**Table 1:** Retrieved models by sample size. The algorithm is able to find the mapping model for the three different data samples. The number of the coefficients remains the same, however, the mean absolute error (MAE) / mean squared error (MSE) values changes. The differences are small are lesser than the size of a pixel (e.g, Δ(200-150) = 0.046, Δ(150-75) = 0.0432). This means that when calculating the gaze position in the camera frame, these differences are not noticeable.

Mapping models	MAE	MSE	Size	Coefficients
9.958 * *x* + 8.00 * *x*^2^ + 0.996 * *x*^3^ − 426.7	0.162	0.042	200	4
9.956 * *x* + 8.001 * *x*^2^ + 0.999 * *x*^3^ − 426.6	0.115	0.023	150	4
9.971 * *x* + 8.000 * *x*^2^ + 0.999 * *x*^3^ − 426.806	0.08	0.010	75	4

The second example, similar to the one shown in [31], used the data generated by the function *f*(*x*) = *sin*(*x*) + 8. We collected 30 pairs (*x*_*i*_, *y*_*i*_). *x*_*i*_ were selected randomly from an interval between 0 and 100, and *y*_*i*_ were obtained by projecting *x*_*i*_ through the function *f*. In addition to the dataset, we gave the following population to the algorithm as input: a list of individuals I={x,cos,log,exp,c∈R} and a list of operators *O* = {−, +, *, /}. Note that the trigonometric sine function, present in f(x) model is not provided in the list of individuals *I*. Here, we seek to assess the behavior of the method when an individual is not included in the list *I*. After 17 milliseconds, the algorithm found the following model:

$$\begin{array}{c} f_{17}\left(x\right)=8-1\cdot \cos\left(4.71-1\cdot x\right) \end{array}$$ (5)

The rapidity of the algorithm depends upon the computer used and the quantity of data which is low in this example (30 pairs). We used the computer described in section *Apparatus and Analysis*. After simplification, *f*_17_(*x*) is similar to *f*(*x*) and can be written as

$$\begin{array}{c} f_{17}\left(x\right)=8-\cos\left(4.71-x+2k\pi \right),k\in Z \end{array}$$ (6)

which can be simplified with simple trigonometric rules to

$$\begin{array}{c} f_{17}\left(x\right)=8-\cos(4.71-x-4\pi /2),\;k=-1 \end{array}$$ (7)

$$\begin{array}{cc} & \approx 8-\cos\left(\frac{3\pi }{2}-x-\frac{4\pi }{2}\right),\;\frac{3\pi }{2}\approx 4.71 \end{array}$$ (8)

$$\begin{array}{cc} & \approx 8-\cos(-\left(\frac{\pi }{2}+x\right)) \end{array}$$ (9)

$$\begin{array}{cc} & \approx 8-\cos\left(\frac{\pi }{2}+x\right),\;cos\left(-x\right)=cos\left(x\right) \end{array}$$ (10)

$$\begin{array}{cc} & \approx 8-\left(-\sin\left(x\right)\right),\;cos\left(\frac{\pi }{2}+x\right)=-sin\left(x\right) \end{array}$$ (11)

$$\begin{array}{cc} & \approx f(x) \end{array}$$ (12)

More examples can be found in [31] and examples using non polynomial models can be found in [34].

### A note on outliers and noise in the dataset

While this method enables the detection of models of previous examples, in real-world scenarios, the collected data can be corrupted by noise and outliers. The noisy data present in real-world come from various aspects altering the measurements. In VOG-based eye tracking systems, eye features detection is prone to error due to the occlusion of eyelashes, eyelids, the presence of mascara, contact lenses, internal defects of cameras or the computer vision algorithm employed [35]. These factors yield false data when collecting calibration points. Considering this fact, we modified 20% (6 observations) of *y*_*i*_ dataset in example 1 in order to simulate noise. The MAE was 1.71 after approximately 200 milliseconds giving a model of the form *f*_200_(*x*) = 9.89*x* + *x*^3^ + 8*x*^2^ − 425. This is in line with our expectations since corrupted records reduce the accuracy of estimators resulting in greater MAE. However, despite the presence of corrupted data (20%), symbolic regression was able to find a reliable model.

### Comparison with polynomial regression

The aim of using polynomial regression is to find solely the coefficients of the mapping function that infers eye features to the planar gaze coordinates. Different mapping models have been proposed in the literature (see [22] for a review), however, considering findings from research that have focused on the models of mapping functions, no model has been shown to provide the best accuracy in all circumstances. For examples in [10], the models that showed good accuracy across 400,000 configurations, on x and y-axis, were:

$$\begin{array}{c} X=a_{0}+a_{1}x+a_{2}y+a_{3}x^{2} \end{array}$$ (13)

$$\begin{array}{c} Y=b_{0}+b_{1}y+b_{2}x^{2}+b_{3}xy+b_{4}x^{2}y \end{array}$$ (14)

and the models that provided the best results, among 625 polynomial models, in [13] were:

$$\begin{array}{c} X=a_{0}+a_{1}x+a_{2}x^{3}+a_{3}y^{2}+a_{4}xy \end{array}$$ (15)

$$\begin{array}{c} Y=b_{0}+b_{1}x+b_{2}x^{2}+b_{3}y+b_{4}y^{2}+b_{5}xy+b_{6}x^{2}y \end{array}$$ (16)

### Extracted models using eye tracking calibration data

**Fig 2:**
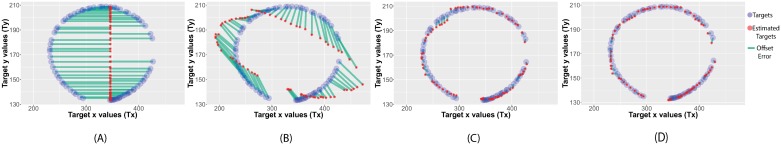
Behavior of the extracted models on the validation points. (a) model at *t*_0_, (b) at *t*_100_, (c) at *t*_500_, (d) at *t*_800_. The fitness quality is represented by the area between the red and violet curves.

Using Symbolic regression, different models are extracted. Some models are less complex than others. Table 2 and Fig 2 present some samples of captured models over time—one participant’s calibration data was tested in this example. The data was smoothed beforehand using 1€ Filter [36], a first-order low-pass filter with an adaptive cutoff frequency. We obtained the models from the training points, as explained in section *Training versus validation points*, and illustrated the examples with validation points. Models on x and y axes are given. Notice that, initially (at *t*_0_), the algorithm starts with either the input values (*P*_*y*_ on y-axis) or a random number lying in the interval of the input values (346 on x-axis). Thereafter, improved models are produced. 500 milliseconds later, the algorithm was able to fit a function using only the x-values of the pupils in the x-axis model. Moreover, a cosine function is used. Non-trivial functions like the cosine function are not easy to guess when deciding on a mapping model a priori in a polynomial regression scenario [13], yet, the algorithm is able to detect and insert them in the models if they foster good accuracy. A combination of the *P*_*x*_ and *P*_*y*_ variables is observed in the model obtained on y-axis. The last models (obtained after 0.8 seconds) have more coefficients and are more complex. Fractions and exponential functions are inserted. The models have an order of up to 4 degrees. We observed that the algorithm did not include the variable *P*_*y*_ in the models on x-axis. As a result, this suggests that a high correlation should exist between the *x* values of the pupil center positions and the *x* values of the marker center positions. Nevertheless, we inspected this statement and proved it in the following section.

**Table 2:** Symbolic regression extracted models.

Time	*t*_0_	*t*_100_	*t*_500_	*t*_800_
Model on x-axis	**G**_**x**_ = 346	**G**_**x**_ = 3.81*x*	$G_{x}=141+0.4\times 10^{-1}p_{x}^{2}+3.3\cdot \cos\left(0.16p_{x}\right)-0.2^{-3}p_{x}^{3}$	$G_{x}=240+1.67p_{x}+(\frac{-4.3\times 10^{7}}{85.7p_{x}+p_{x}^{3}-17.1p_{x}^{2}})+(\frac{1.7\times 10^{9}}{p_{x}^{4}+85.7p_{x}^{2}-17.1p_{x}^{3}})$
Model on y-axis	**G**_**y**_ = *y*	**G**_**y**_ = 2.19*y* − 166	$G_{y}=3.0p_{y}+0.03p_{x}^{2}-270.1-2.4p_{x}-0.14\times 10^{-3}p_{x}^{3}$	**G**_**y**_ = 35.5*py* + 0.003*px*^2^ + 2.2 × 10^5^ ⋅ exp(−0.04*py*) − 4.6 × 10^3^ − 1.54 × 10^−6^ *py*^4^ − 6.5 × 10^5^ exp(−0.21*px*)

### Pearson’s product-moment correlation coefficient

**Fig 3:**
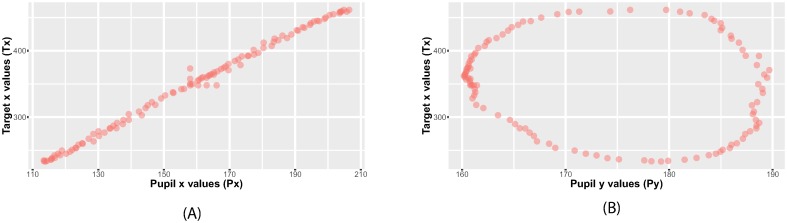
Pearson’s correlation. (a) A scatter plot indicating the strong positive linear relationship between the x-values of pupil data and the x-values of the target (*r* = 0.9958, *p* < .0001). (b) There is no linear relationship between the y-values of pupil data and the x-values of the target (*r* = −0.5557, *p* < .0001).

The Pearson’s correlation coefficient is a measurement of the strength of the linear correlation between two variables. There is a positive correlation if both datasets fall or rise together in the same direction. [37] applied Pearson’s product-moment correlation on eye tracking data. They used it to select among many targets, the one at which the user is looking. Esteves et al. [38] designed a method of selecting widgets on a smartwatch based on the correlation between moving targets and the user’s eye positions. Those approaches inspired many subsequent studies and Pearson’s correlation proved to work well for eye tracking data [39, 40]. To validate our statement in section Extracted models using eye tracking calibration data, that is, there are more *x* individuals than *y* in the obtained model *f*_*x*_ because the *x* values of the pupil are more closely correlated to the *x* values of the marker centers, we used Pearson’s correlation. As a result, the correlation between the x-values of pupil data (*p*_*x*_) and the x-values of the target (*f*_*x*_) was *r* = 0.9958, *p* < .0001 confirming a strong linear relationship as shown in Fig 3A, while the correlation between the y-values of pupil data (*p*_*y*_) and target (*f*_*x*_) was *r* = −0.5557, *p* < .0001 (Fig 3B).

### Applying symbolic regression on different calibration patterns

**Fig 4:**
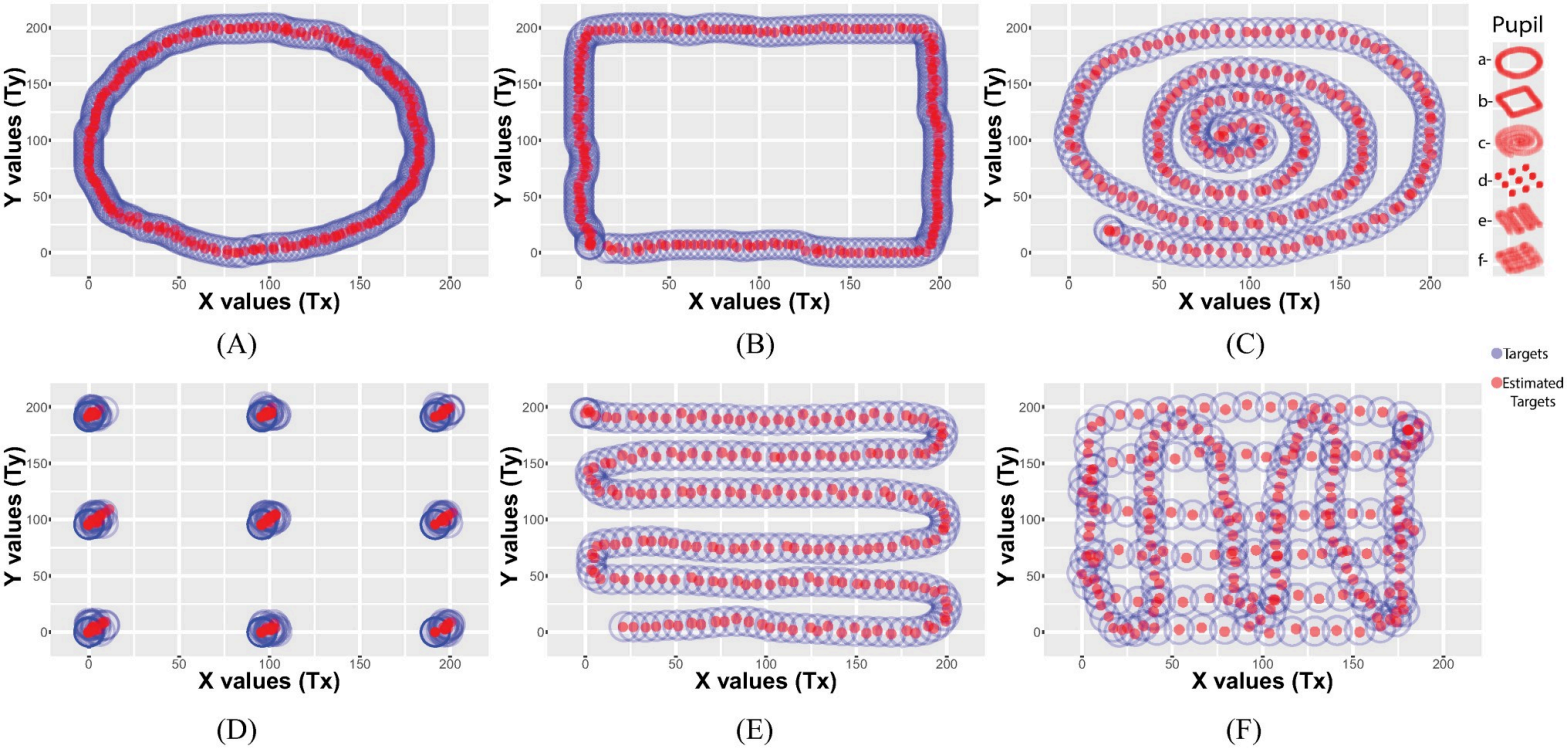
Fitted models obtained using symbolic regression on 6 different calibration patterns. The blue circles represent the training points and the red circles represent the estimated points. Symbolic regression was able to find reliable models that map the pupil positions to the target positions for the 6 calibration patterns.

**Fig 5:**
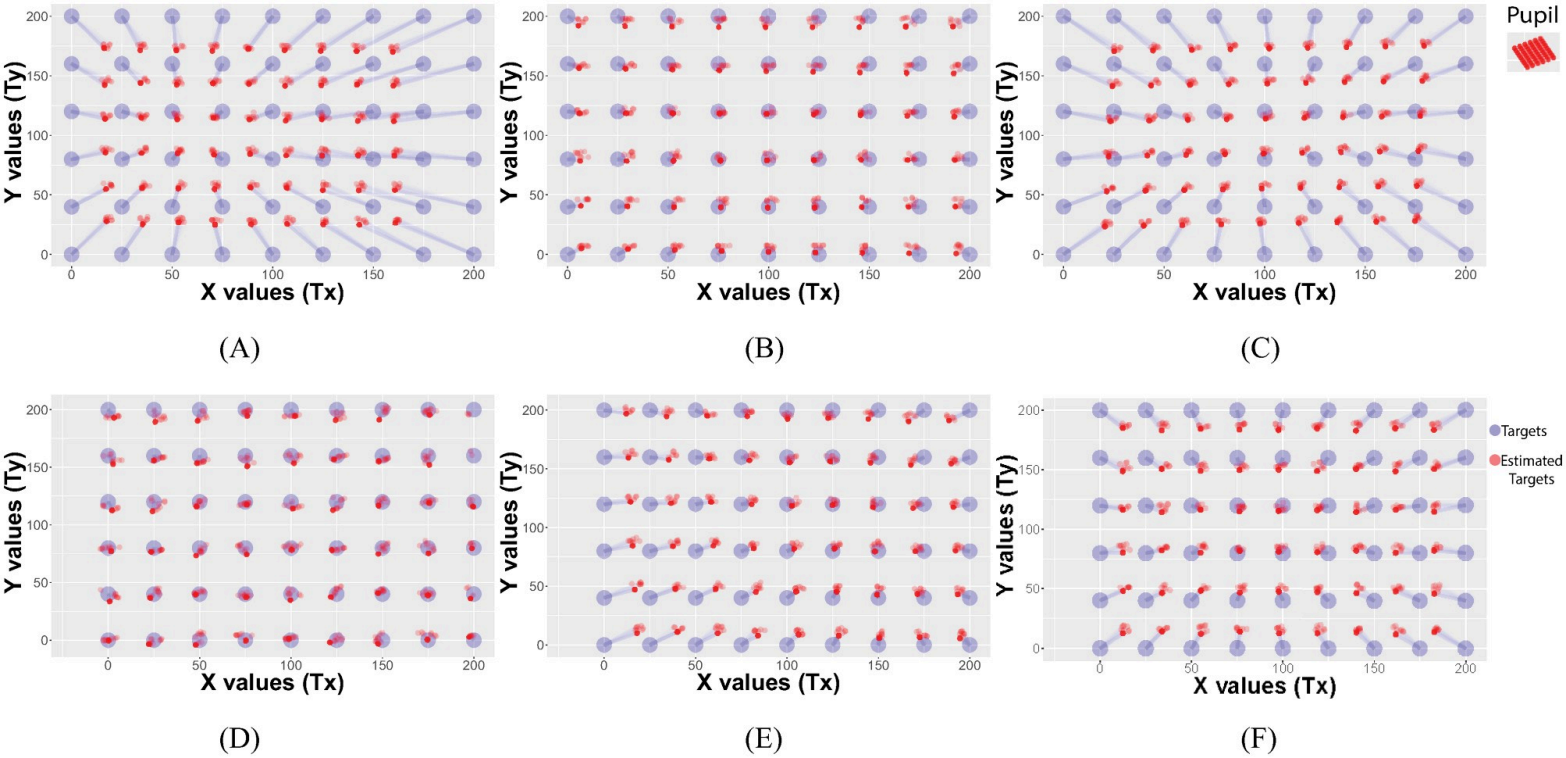
Reprojection of the validation points (9×6 lattice points) using the models obtained from the symbolic regression algorithm on the 6 calibration patterns. The circular and spiral calibration patterns (a. and c.) resulted in lower accuracy compared to the 9-point and rectangular calibration patterns (b. and d.).

To assess the applicability of symbolic regression on the different calibration patterns, we considered six approaches proposed in the literature and used symbolic regression to find the mapping models as shown in Fig 4. The accuracy of the models was evaluated with a lattice defined by 9×6 points covering the whole screen (Fig 5). Overall, the figures clearly indicated that the method was able to find reliable models for each pattern. However, while the estimated points are close to the training points in Fig 4, we also observe a significant displacement between the true lattice points position and the estimated lattice points position (Fig 5). In particular, the circular and spiral calibration patterns (Fig 5a and 5c) resulted in lower accuracy compared to the 9-point and rectangular calibration patterns (Fig 5b and 5d).

## Method: Evaluation of SP and VOR calibration using symbolic regression

### Smooth pursuit calibration

**Fig 6:**
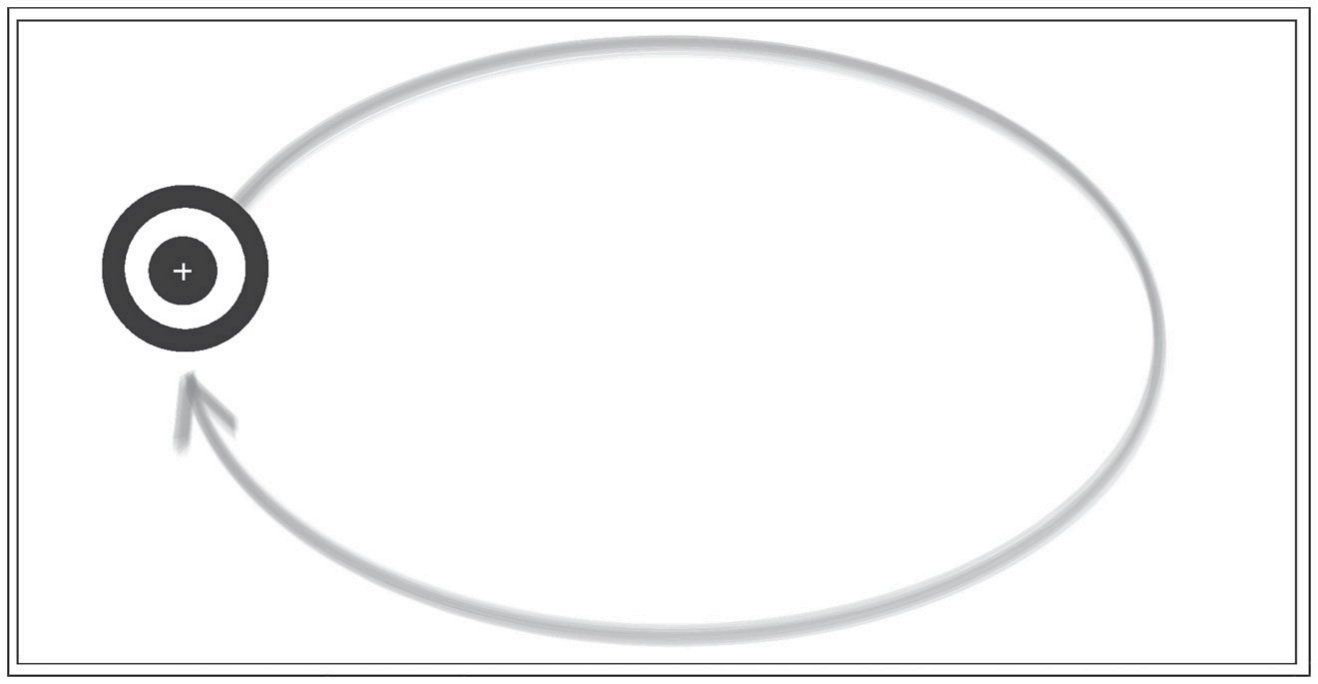
Illustration of the marker used in Study 1. A white cross is drawn on the center of the marker and corresponds to the point the user is requested to fixate on during the calibration. The curve printed in the gray stroke corresponds to the pattern followed by the marker. It is represented here for illustration purposes and is not visible on the monitor.

SP calibration is a satisfying alternative for subjects having difficulties fixating on static targets and maintaining concentration over a long period [15]. To test this calibration routine, a marker was designed to follow a predefined trajectory on a screen. We asked the participants to keep their head still and to stare at the moving marker during the calibration. The trajectory of the marker, shown in Fig 6 was arranged so that it covered a large area of the screen.

### Vestibulo-ocular reflex calibration

Vestibulo-ocular reflex calibration (VOR) is of interest where there is no system enabling the movement of the marker. For instance, calibrating an eye tracker in an outside environment. In the experiment, we printed a fixed marker and asked the participant to fixate on the marker while rotating her head. In this manner, we were able to collect different positions of the fixed marker in the world camera reference system. The coverage of the marker positions in the camera and the speed of the head movement depended on the participant.

### Target types: Marker vs. finger

The calibration procedure described so far uses markers as a target. Such an arrangement is only possible when a marker is available. When conceiving an eye tracking system, some questions might be raised, for example, how could a calibration be easily performed alone and without any marker. In some situations, an assistant can hold the marker in front of the user but this implies additional human involvement each time a new calibration is needed. The user’s fingertip can be used to mimic the target instead. Finger-as-target is one substitute for calibration without the presence of a marker. A finger is adequate for cases in which we have no object to use as a marker and we have no personal assistance [41]. Consequently, gathering all the mentioned factors, we investigated and provide results for four different calibration procedures, i.e, SP calibration with marker, SP calibration with finger, VOR calibration with marker and VOR calibration with finger.

The majority of these calibration procedures have already been tested separately [8, 9]. In this study, we used a monocular eye tracker which is an affordable system with only one eye camera and a world camera, in contrast to the frequently studied binocular systems [4].

### Data collection

The data were recorded as (x,y) position coordinates for all participants. The pupil center positions were detected in the eye camera frame and the target positions in the world camera frame. Lighting conditions were controlled to be the same throughout the two experiments. The experimenter started and ended the data collection by pressing a button. No filtering was applied during the collection. We recorded the data at an average rate of 114 frames per second. Each frame allowed the detection of one or zero points (zero if there is no detection). For each calibration, we excluded the duplicated pupil and marker centers entries and their corresponding pairs. We intentionally left a few milliseconds before the marker actually starts moving to ascertain that the data samples are effectively recorded from this initial position. Therefore, 5% of the starting and 5% ending points were removed for each participant. The last 5% points were removed to deal with the data points corresponding to the moment the marker stops moving and the moment the data recording is effectively stopped by the experiment facilitator. Outliers were removed and the collected data were normalized before the regression. The study was approved by the local committee at the French Civil Aviation University. The goal was only to record a one minute video of participants’ eyes and fill a questionnaire. Written informed consent forms were signed by all participants and all the experiment tasks were performed at the French Civil Aviation University.

### Training versus validation points

We used a cross-validation method to evaluate the accuracy of the models [19]. The collected data obtained during the calibration procedure were divided into two separate datasets, the training (60%) and validation (40%) sets. The first set was used to find a mapping function through regression and the second set served to compute the gaze estimation. In other words, it allowed the calculation of the reprojection errors corresponding to the offset between the estimated and the measured value [42]. The reprojection errors were used to compute the calibration accuracy, i.e., the closeness with which the measurement of the indicated gaze estimation is related to the actual target position. High accuracy means that the estimation is on or very close to the target position.

## Study 1: Marker-as-a-target calibration

The first study dealt with calibration procedures using a marker. Through a series of tasks, we explored the difference in results between the calibration procedures described in section and, both using a marker as a target, and the effect of Symbolic regression on the mean absolute error (MAE). The participants performed the calibration procedures and all analyses were made after the experiment. To this end, only calibration data were collected during the experiment which spanned two days.

### Method

**Participants**: 6 participants were recruited for this experiment, making a particular attempt to include participants having different qualifications and educational levels ranging from high school to postgraduate level. Half of the participants (3) were women aged from 17 to 27 years old and the others were men aged from 22 to 30 years old. Upon arrival, they were asked to sign a consent form and demographic information was retrieved. The completed forms showed that 2 participants were familiar with eye tracking systems.

**Marker detection**: During the stage of user calibration phase, the participant is asked to fixate on a reference point, represented by the center of a marker in this study (Fig 6). the choice of the marker has been a well-studied problem [8]. A simple marker whose shape is not confused with any other object in the room is appropriate; the marker must not have many details so as not to distract the participant and its center must easily be computable with affordable computer vision techniques. A pilot test showed that there is no accuracy, precision or time difference when using a different marker, as expected. Each participant performed the calibration routines and after that, the first calibration performed by the participant was repeated to assess possible fatigue effect caused by the duration of the study. In our study, the marker consists of a thick black circle containing a white circle which in turn encompasses a smaller filled black circle drawn on a white background, similar to the markers used by Tobii and Pupil Labs (Fig 6). A white cross is drawn on the center of the marker.

**Trajectory of the marker**: Different paths have been tested in recent studies. [1] used a rectangular path, varying the speed of the moving target. [9], [8] and [16] considered spiral paths, while [17] employed a star pattern. [15] studied a vertical and horizontal path for difficult to calibrate participants. Since, to our knowledge, there is no study that has proved empirically which path provides more accurate results, we considered a circular smooth trajectory starting from the left and following a complete circle as shown in Fig 6. This path has the advantage of circumventing the problem of abrupt changes in direction elucidated in [1]. In this case, the marker could move smoothly with constant velocity. In addition to the simplicity of the path, the calibration takes less time compared to a calibration using a spiral path. This is important because it reduces the effects of fatigue during the calibration procedure.

### Apparatus and analysis

**Fig 7:**
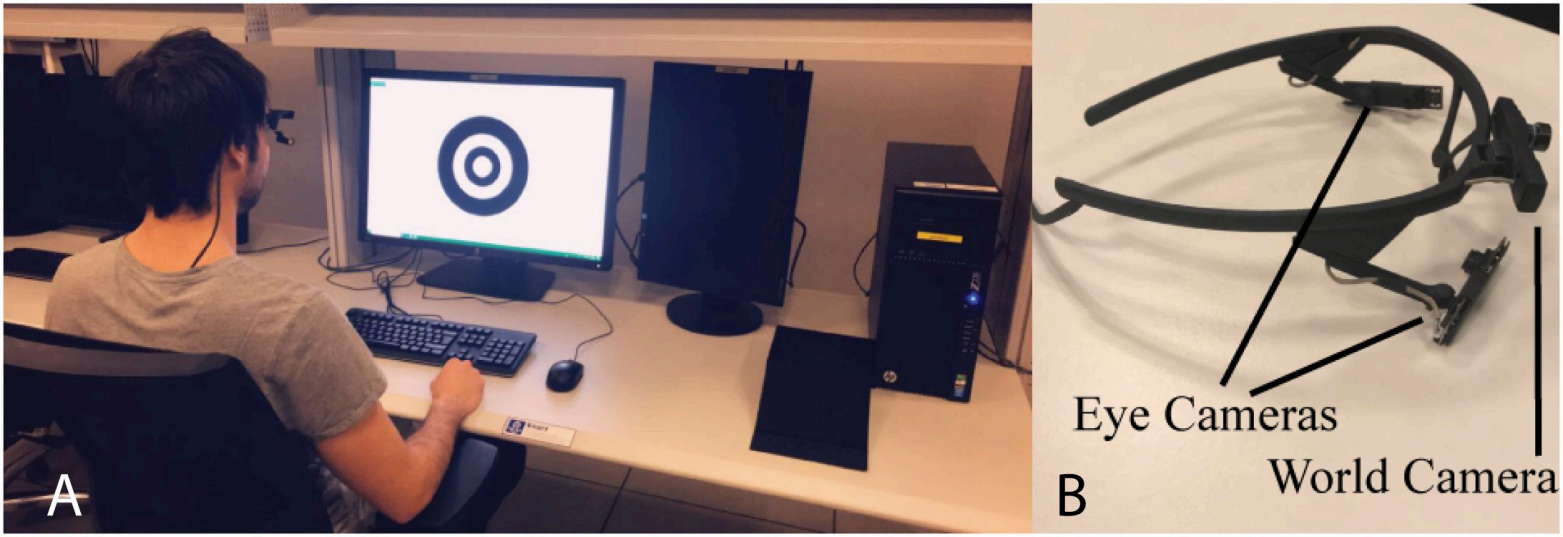
Setup and apparatus. Illustration of (A) the experimental setup and (B) the Pupil Labs eye tracking system. One eye camera was used during the study.

Fig 7B depicts the eye tracking system. A Pupil Labs Eye tracker was used during the two experiments. The device is equipped with one world camera (Sampling rate: @120Hz, 1920X1080 pixels) and one eye camera (Sampling rate: @120Hz, 640X480 pixels). The data collection was processed at 114 Hz.

A C# desktop software was built with EmguCv 3.1 for the computer vision technique implementation. The equipment setup was a XPS 15 9530 Dell Laptop ×64, Intel Core I7-4712HQ CPU@2.30GHz, 2.3 GHz, 16 GB of Random Access Memory, 2GB swapping Memory. We used a 24 inch Dell 2408 WFP monitor (L x W x H Dimensions: 22 x 8.17 x 15.62 inches) which has a resolution of 1920x1200 pixels and 24 Milliseconds response time. The marker was displayed in the monitor which is placed 75 cm from the participants. The experimental setup is illustrated in Fig 7A.

In this experiment, two hypotheses were tested. Initially, We tested the hypothesis that the mean difference between smooth pursuit (SP) and vestibulo-ocular reflex (VOR) calibration using standard regression is significant. Then, we checked if the mean difference between the accuracies obtained using symbolic regression and standard polynomial regression is significant for SP and VOR calibration separately. A paired T-test was required to determine whether the differences were significant using **R**. We set an alpha level of.05 for all statistical tests.

### Results

**SP vs VOR calibrations using standard regression**: The data of all participants were included in the analysis. A paired T-test showed that there was no significant difference in accuracy between SP (*M* = 0.81, *SD* = 0.21) and VOR (*M* = 1.4, *SD* = 1.23) calibration on x axis, *t*(5) = 1.10, *p* = 0.3. However, SP calibration resulted in better results compared to the VOR calibration on y axis (*t*(5) = 3.161, *p* = 0.025). The Mean of the differences observed was 0.49°. These results suggested that SP calibration can help reduce gaze estimation accuracy on at least one component of Cartesian coordinates plane, that is, y axis in this example (Table 3).

**Table 3:** MAE. Mean Absolute Error and Standard Deviation of SP and VOR calibrations based on marker, on x and y axes, using standard regression.

	Smooth Pursuit		Vest. Ocular R.	
	x	y	x	y
*μ*(°)	0.813	0.441	1.404	0.937
*σ*(°)	0.212	0.146	1.233	0.331

**SP using standard regression vs SP using symbolic regression**: Although the results shown in Table 4 bear witness to the success of the symbolic regression algorithm in reducing estimation errors and increasing accuracy, it was still valuable to statistically evaluate whether the differences were significant. Descriptive analysis showed the effect of symbolic regression in reducing the mean absolute error after only 1-second search, resulting in a mean absolute error of 0.69° and 0.313° on *x* and *y* axes respectively. After 10 seconds of searching, the absolute error was reduced to 0.67° and 0.27°. A paired t-test was conducted between smooth pursuit calibration using symbolic regression and smooth pursuit calibration using standard polynomial regression. The same data was used for both algorithms. Results showed a significant main effect of symbolic regression over the commonly used polynomial regression, both on *x*(*t*(5) = 5.07, *p* < .05) and *y* axes (*t*(5) = 3.79, *p* < .05).

**Table 4:** MAE. Mean Absolute Error and Standard Deviation of SP and VOR calibrations based on marker, on x and y axes, using symbolic regressions after 1s, 10s and 30s.

	Smooth Pursuit		Vest. Ocular R.	
	x	y	x	y
***μ*(°)(1*s*)**	**0.696**	**0.313**	**0.889**	**0.715**
*μ*(°)(10*s*)	0.672	0.272	0.856	0.680
*μ*(°)(30*s*)	0.664	0.269	0.818	0.666
***σ*(°)(1*s*)**	**0.180**	**0.132**	**0.785**	**0.371**
*σ*(°)(10*s*)	0.158	0.108	0.753	0.365
*σ*(°)(30*s*)	0.161	0.108	0.675	0.339

**VOR using standard regression vs VOR using symbolic regression**: Comparisons between vestibulo-ocular calibration using the standard regression and vestibulo-ocular calibration using symbolic regression yielded significant differences. Results showed that after 1 second of search, symbolic regression was able to establish better estimations on both *x*(*t*(5) = 2.69, *p* < .05) and *y* axes (*t*(5) = 3.41, *p* < .05). Descriptive analysis of the results obtained with symbolic regression after 10 seconds and 30 seconds are given in Table 4.

## Study 2: Finger-as-a-target calibration

The second study concerned the calibration procedures based on using the finger as a target. The goal of the study was to assess the effect of the proposed symbolic regression approach on recent calibration methods [41]. As in the previous study, the post-processing and calculations were performed after the data gathering. Here, we investigated the impact of letting the participant decide the locations and the movement of the target on the gaze estimation accuracy.

### Method

**Participants**: 6 participants(2 males and 4 females) aged from 23 to 32 took part in the experiments. 3 participants wore glasses during the experiments. The purpose of the study was explained to the participants, thereafter they signed a consent form.

**Fig 8:**
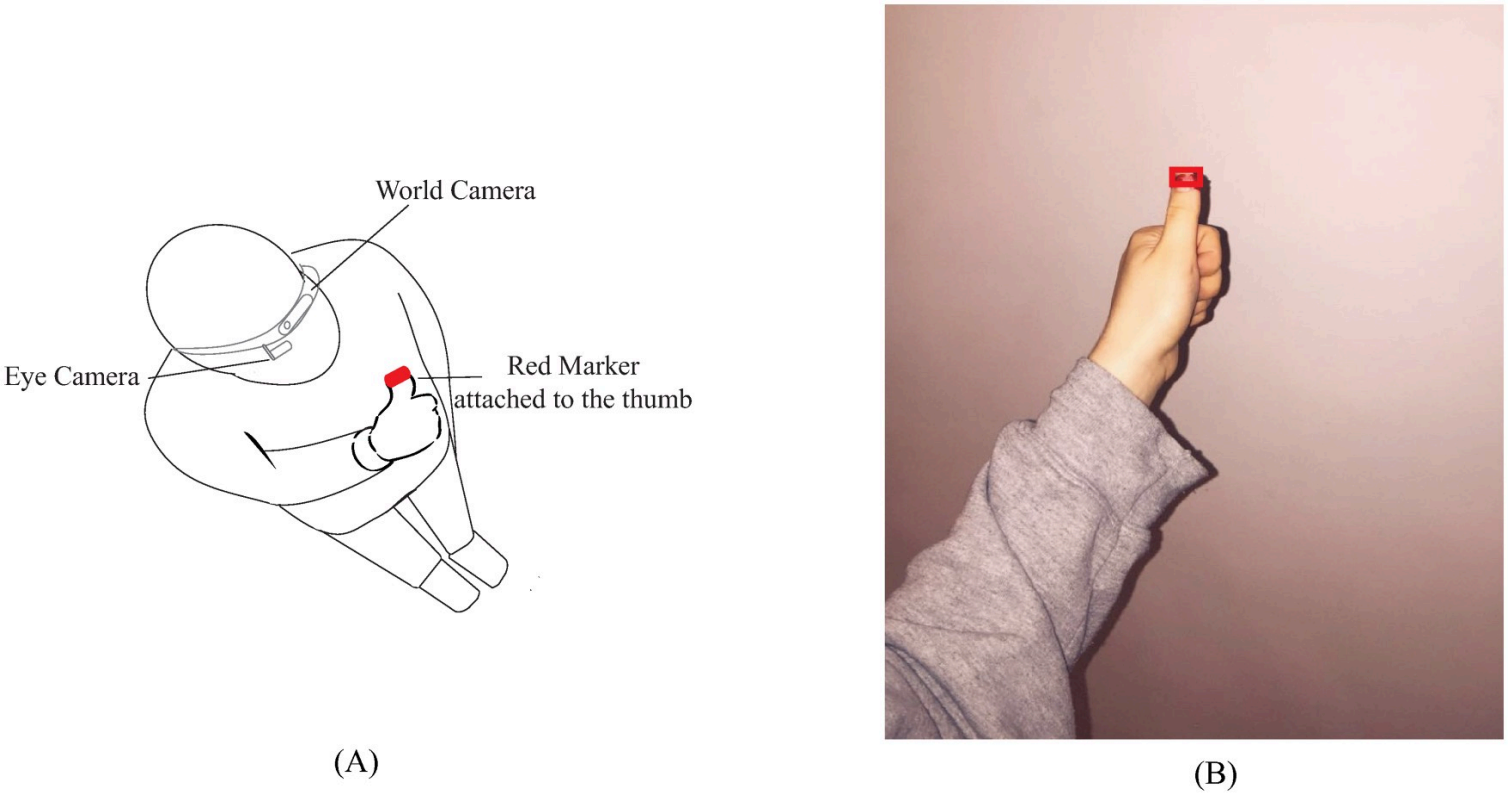
Finger calibration. (a) A user performing a calibration using her thumb as a target. A red marker is attached to the tip of her thumb to allow easy detection of her thumb using computer vision techniques. (b) Illustration of a frame obtained in the world camera video stream.

**Finger detection**: The idea of using a finger as a marker comes from the implementation of the Sixth Sense wearable prototype [43]. While Sixth Sense relies on different coloured markers attached to the fingers to enable interaction using hand gestures, in our study, a similar red marker was attached to the user’s thumb and was used as a target that enables collecting gaze points in the world camera view, as illustrated in Fig 8. More precisely, the user was required to attach a red marker to the tip of her thumb and fixate on the top left corner of her thumb during the calibration procedure. An algorithm processes the world camera stream and tracks the user’s finger using computer-vision algorithms [43].

**Procedure and Task**: The study was structured as a within-subjects design wherein each participant completed the two calibration procedures requiring the finger as a target. Each participant was asked to rotate her head while maintaining her thumb still for vestibular movements calibration. For smooth pursuit calibration, the participant was asked to keep her head still, while rotating and making a smooth circle trajectory with her thumb. We asked participants to try their best doing the task as calibration may be unusual in terms of the coordination of head or finger movements due to natural human dynamics.

### Apparatus

The same apparatus described in study 1 was used, except for the monitor because a marker is no longer needed in this configuration. Instead, a red marker, carefully attached to the user’s fingertip, as exemplified in Fig 8 was used as a target.

### Results

A dependent t-test was conducted to compare the effect of calibrating with SP or VOR movements using standard regression. There was no significant effect on *x* (*t*(5) = 1.28, *p* = 0.25) and *y* axis (*t*(5) = 0.71, *p* = .5). However, as in study 1, symbolic regression was able to find a model that reduced the error 1 second after the beginning of the search and statistically significant differences were observed between SP calibration using standard and SP calibration using symbolic regression on *x* (*t*(5) = 3.32, *p* < .05) and *y* axes (*t*(5) = 2.8, *p* < .05). Nonetheless, while the same significance was observed for VOR calibration using standard and VOR calibration using symbolic regression on *y* axis (*t*(5) = 2.59, *p* < .05), no significant difference was found on *x* axis (*t*(5) = 2.21, *p* = 0.705). We provide the means and standard deviation obtained with standard and symbolic regression respectively in Tables 5 and 6.

**Table 5:** MAE. Mean Absolute Error and Standard Deviation of SP and VOR calibrations based on finger, on x and y axes, using standard regression.

	Smooth Pursuit		Vest. Ocular R.	
	x	y	x	y
*μ*(°)	1.045	1.087	1.788	1.362
*σ*(°)	0.234	0.297	1.423	0.869

**Table 6:** MAE. Mean Absolute Error and Standard Deviation of SP and VOR calibrations with finger, on x and y axes, based on symbolic regressions after 1s, 10s and 30s.

	Smooth Pursuit		Vest. Ocular R.	
	x	y	x	y
***μ*(°)(1*s*)**	**0.714**	**0.658**	**0.971**	**0.738**
*μ*(°)(10*s*)	0.651	0.652	0.851	0.717
*μ*(°)(30*s*)	0.646	0.640	0.777	0.714
***σ*(°)(1*s*)**	**0.396**	**0.272**	**0.549**	**0.330**
*σ*(°)(10*s*)	0.318	0.267	0.373	0.329
*σ*(°)(30*s*)	0.314	0.245	0.344	0.329

## Discussion

Each of the considered methods has different advantages and downsides. In particular, SP is the easiest to perform and allows for data collection in the region where the designer wants the calibration to be the most accurate since it requires a moving marker with a predefined path. However, it has the disadvantage of being extremely sensitive to hardware synchronization, the anticipation of the marker’s path by the user, and requires additional support that enables the change of the marker’s position, for example, computer or human assistance.

Vestibulo-ocular reflex movements calibration allows an adaptation to the natural behavior of participants that has long been considered as a constraint during the calibration, namely the movement of the head which results in poor data quality. In some studies, researchers always use a chin-rest to lessen the small head drifts [44]. Its major disadvantage is that it requires additional user implication during the calibration. Hence its application is not suited for eye trackers that are susceptible to slip or move.

**Fig 9:**
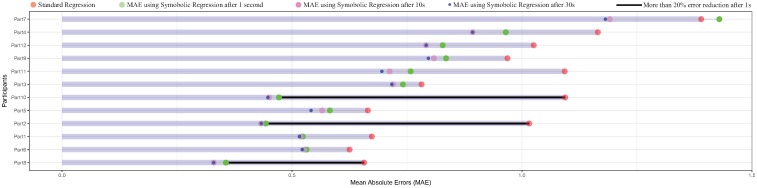
Reduction of the mean absolute error (MAE) in time for all participant on x-axis. A similar effect is observed for y-axis. The MAE obtained using the standard regression (orange circles) decreased significantly when using symbolic regression. It is clearly visible that the MAE obtained after 1 second (green circles) can help reducing gaze estimation accuracy in real time scenarios. For some participants, the difference between symbolic and standard regression errors (represented in bold black line) exceeded 50% (participants 10, 2 and 8). Also, note that for participant 7, the algorithm did not find directly a better model after 1 second, but a few moments later.

Overall, symbolic regression was able to improve gaze estimation accuracy for all participants. Fig 9 shows to what extent symbolic reduction decreased the mean absolute errors for all participants. For clarity purposes, only data on x-axis and for finger calibration were reported. However, similar effects were observed on y-axis and for marker-based calibrations.

**Fig 10:**
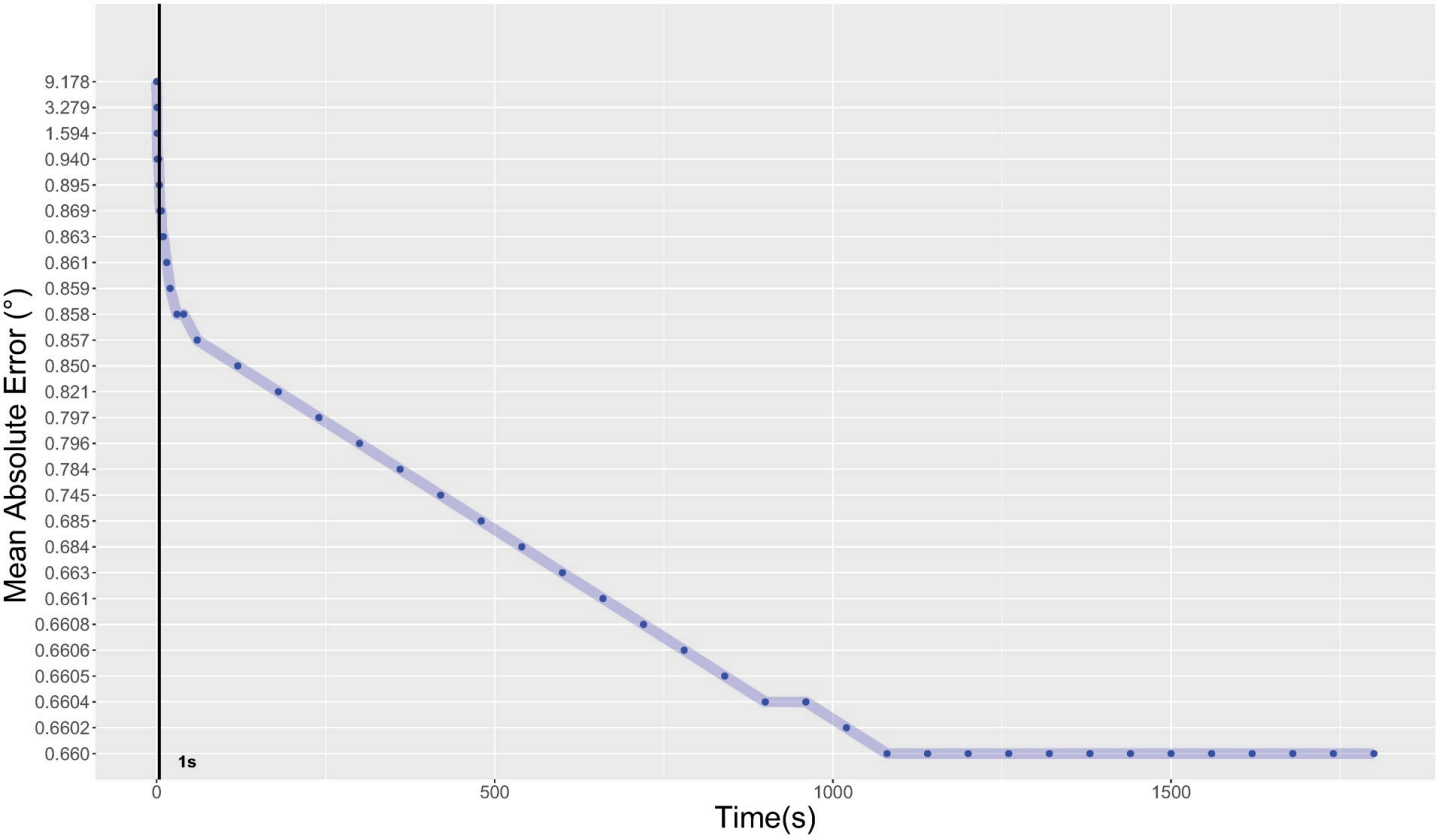
Reduction of the MAE over time using symbolic regression for one participant. The algorithm stabilizes after a few milliseconds and no better model is extracted.

Fig 10 shows the behavior of symbolic regression applied to the data obtained from one participant. Initially, the error models obtained gave an error of 9.17° (at *t*_0_). The next model obtained after 500 milliseconds gave an error of 3.27°. Suddenly, the error decreased to 0.89° after 1 second and 15 seconds later, the error was reduced to 0.85°. The algorithm kept returning models that were increasingly accurate. We deliberately let the algorithm pursue the research and found that after a certain amount of time (18 minutes for this example), the method no longer provides better results. We can conclude that letting the method searches models over a long time does not necessarily help improving accuracy, however, a complete study should be performed to investigate this statement. Nevertheless, if the designers want to get the best model while keeping a real-time process of their application, the model obtained after a few milliseconds could be used and the algorithm could continue seeking better models in the background, replacing the current model each time a better one is extracted.

## Conclusion

In this paper, we investigated symbolic regression to improve eye tracking system calibration accuracy. We validated the methods by comparing results obtained from models using standard polynomial regressions and symbolic regressions. We collected 48 (12 × 4) trials with 12 participants and investigated 4 different calibration procedures: Smooth Pursuit and Vestibulo-Ocular, both using the finger and a marker as a target. Thorough analysis of the collected data was performed and statistical results showed the benefit of symbolic regression on calibration accuracy with a reasonable extra computation time.

This paper presents the first investigation of symbolic regression with monocular eye tracking system calibration. This type of computation opens up promising perspectives in terms of flexibility and accuracy. This work can be directly extended with binocular calibration (one symbolic regression per eye). While the presented method improved standard calibration, additional work should be performed to compare it to other calibration methods such as the one based on eye geometry. As future works, we plan to extend the symbolic regression method with a predefined set of functions in order to make the system converge faster with an accurate regression. We also plan to test this new calibration with different types of eye trackers: table based eye tracker, immersive and mix reality devices.

## Supporting information

S1 File This dataset contains the calibration error per participant in *x*- and *y*-axis for marker calibration using standard regression. The data were used to prepare Table 3.
(XLSX)

S2 File This dataset contains the calibration error per participant in *x*- and *y*-axis for marker calibration using symbolic regression after 1s, 10s, and 30s. The data were used to prepare Table 4.
(XLSX)

S3 File This dataset contains the calibration error per participant in *x*- and *y*-axis for finger calibration using standard regression. The data were used to prepare Table 5.
(XLSX)

S4 File This dataset contains the calibration error per participant in *x*- and *y*-axis for finger calibration using symbolic regression after 1s, 10s, and 30s. The data were used to prepare Table 6.
(XLSX)
